# Adult stem cell sources for skeletal and smooth muscle tissue engineering

**DOI:** 10.1186/s13287-022-02835-x

**Published:** 2022-04-11

**Authors:** Souzan Salemi, Jenny A. Prange, Valentin Baumgartner, Deana Mohr-Haralampieva, Daniel Eberli

**Affiliations:** Laboratory for Urologic Oncology and Stem Cell Therapy, Department of Urology, University Hospital Zürich, Wagistrasse 21, 4.OG, 8952 Schlieren, Switzerland

**Keywords:** Adult stem cells, Tissue engineering, Skeletal muscle, Smooth muscle

## Abstract

**Introduction:**

Tissue engineering is an innovative field with enormous developments in recent years. These advances are not only in the understanding of how stem cells can be isolated, cultured and manipulated but also in their potential for clinical applications. Thus, tissue engineering when applied to skeletal and smooth muscle cells is an area that bears high benefit for patients with muscular diseases or damage. Most of the recent research has been focused on use of adult stem cells. These cells have the ability to rejuvenate and repair damaged tissues and can be derived from different organs and tissue sources. Recently there are several different types of adult stem cells, which have the potential to function as a cell source for tissue engineering of skeletal and smooth muscles. However, to build neo‐tissues there are several challenges which have to be addressed, such as the selection of the most suitable stem cell type, isolation techniques, gaining control over its differentiation and proliferation process.

**Conclusion:**

The usage of adult stem cells for muscle engineering applications is promising. Here, we summarize the status of research on the use of adult stem cells for cell transplantation in experimental animals and humans. In particular, the application of skeletal and smooth muscle engineering in pre-clinical and clinical trials will be discussed.

## Introduction

There is a high clinical demand for tissue-engineered skeletal and smooth muscles for transplantation or replacement therapy. Tissue engineering (TE) approaches would be indispensable in treating diseases that affect skeletal and smooth muscles, including cases of muscular dystrophies, volumetric muscle loss after cancer or trauma and aging. Muscle tissues that require contractile activity for proper functioning, could also be repaired or replaced by the means of TE, such as the various sphincters, bladder, intestine, diaphragm, face, hand, tongue, pharynx, larynx and oesophagus. In general, TE uses progenitor cells in combination with suitable biomaterials that together generate the appropriate microenvironment to functionally repair, replace and regenerate the damaged or lost organ [1, 2]. However, production of engineered tissues and organs requires the use of large number of cells. But there are several problems using the patients` own cells, such as cancer, complex surgery for access, biopsy size limitation, especially in the pediatric population, harvest site morbidity, and low cell proliferation potential. TE using adult stem cells (ASCs) offers a feasible solution to these problems, and paves the road to biological substitutes that can help to restore, maintain or improve tissue function [3]. ASCs are derived from postnatal tissues, including fetal derived stem cells and umbilical cord blood stem cells. The ability of ASC to divide or self-renew makes them an attractive cell source for use in TE. Recently, ASCs have been isolated from every tissue and organ type in mammals [4]. ASCs with their distinctive plasticity have the ability to rejuvenate and repair damaged tissues and organs when transplanted in human [3]. Tumorigenicity and ethical concerns have hindered the widespread use of embryonic stem cells in clinical applications. Therefore, ASCs have stirred a much greater interest for use in regenerative medicine and are being tested and approved for several clinical applications [5, 6].

ASC can be used for repair or cell replacement in patients with a variety of muscular diseases. Muscles are mainly responsible for maintaining and altering posture, locomotion, movement of internal organs, such as the contractions of the heart, sphincter and bladder, and the bowel movement. Muscle tissue is a mesodermal soft tissue, which is formed during embryonic development over a process of myogenesis. There are three types of muscles: skeletal (striated), cardiac, and smooth muscle. This review focuses on the use of different cell types for engineering of skeletal and smooth muscles, with emphasis on the use of autologous ASCs from various sources.

**Fig. 1:**
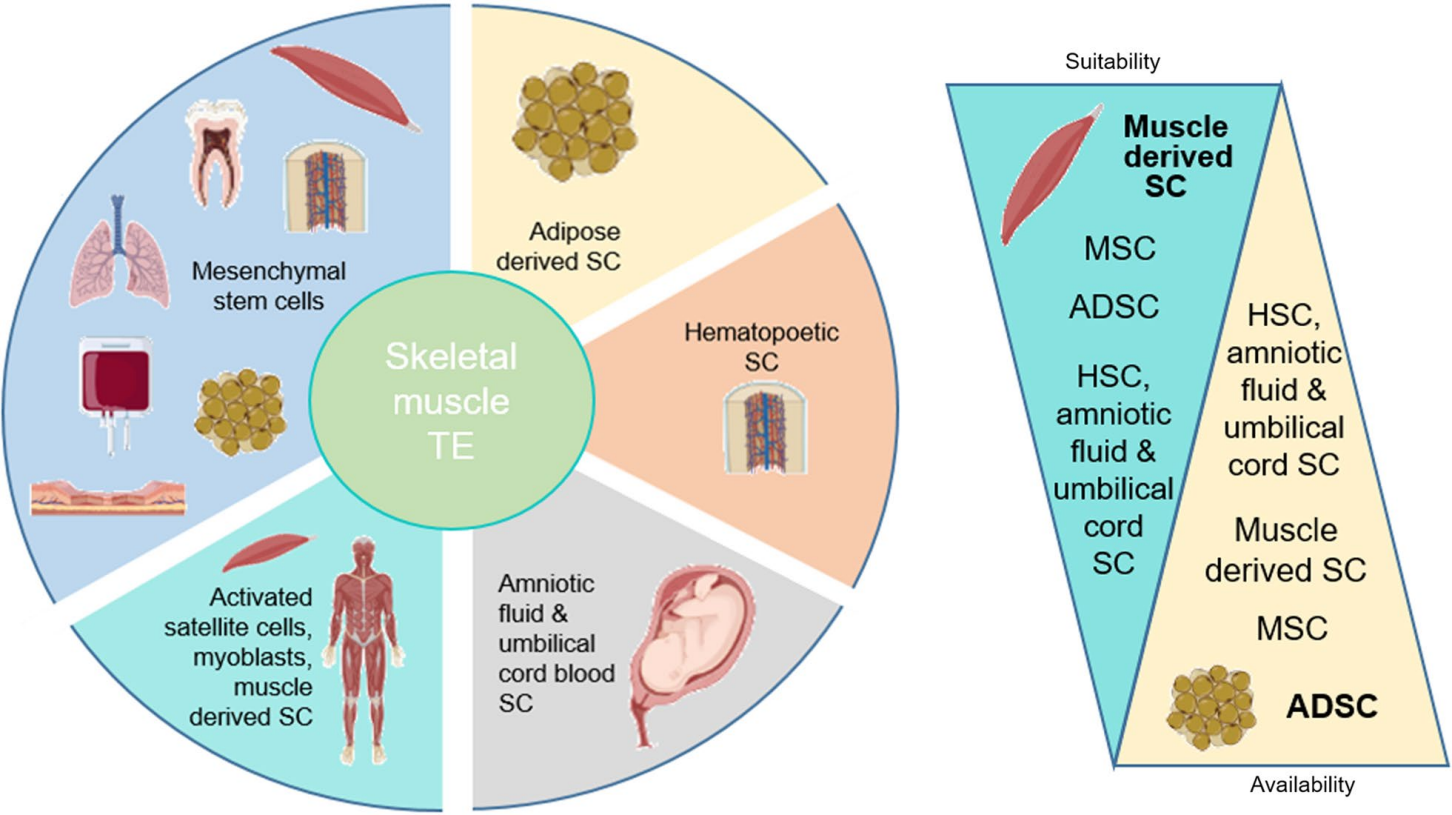
Schematic presentation of potential adult stem cells for skeletal muscle tissue engineering icons were generated using Biorender

**Fig. 2:**
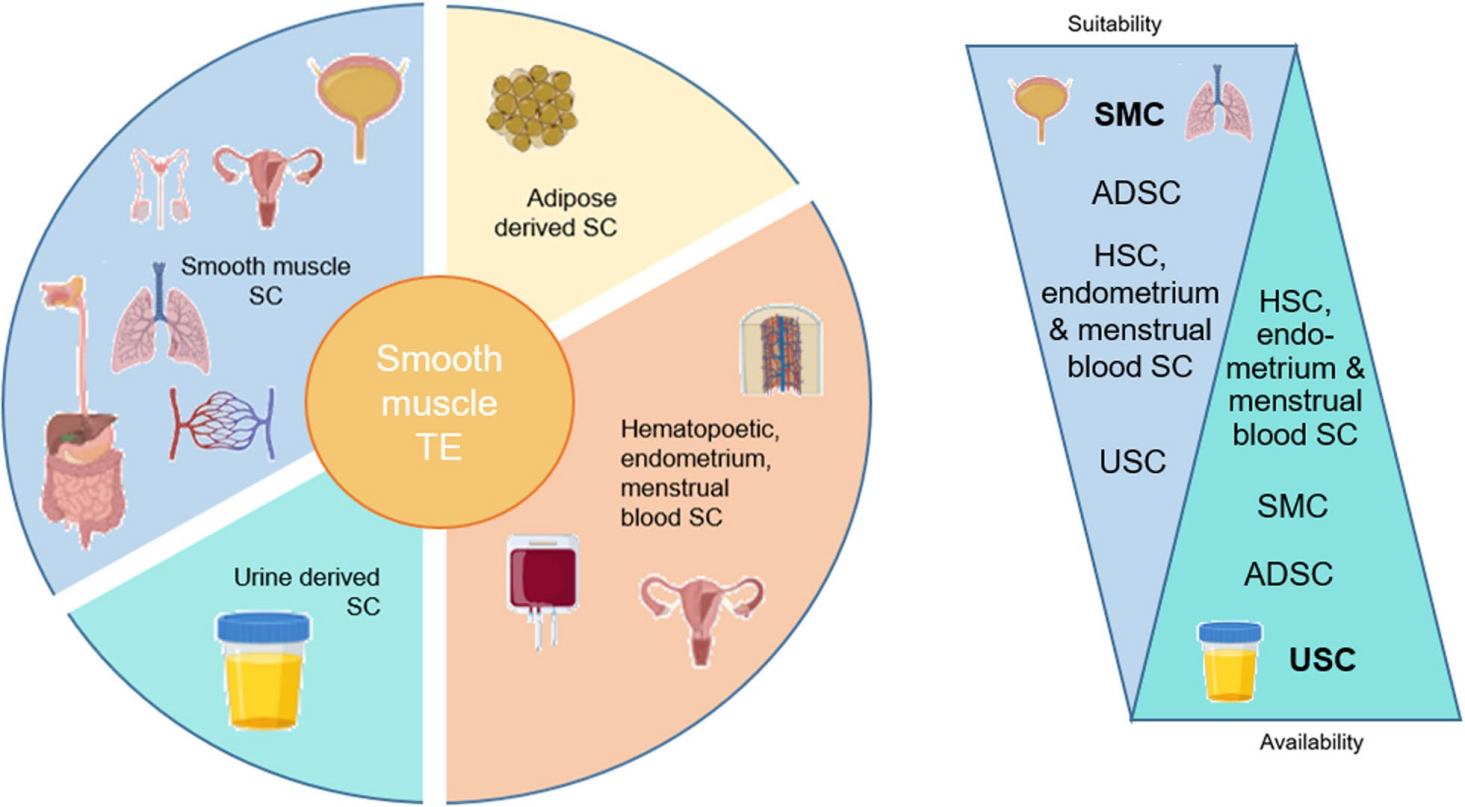
Schematic presentation of potential adult stem cells for smooth muscle tissue engineering icons were generated using Biorender

The purpose of creating clinically-relevant engineered tissue places specific requirements on the cell type and the source, including accessibility with minimal invasiveness, the ability to produce large number of cells in a short time period and minimal changes during in vitro processing, differentiation potential and reproducibility. These considerations have favored certain stem cell types over the others. A significant amount of research has shown the possibility that ASCs are the therapeutic alternative to embryonic stem cells because of their plasticity [7]. Here, we summarize the status of research on cell transplantation in experimental animals and humans. We focus on stem cells that have been tested for skeletal and smooth muscle therapies. The success of engineered muscle tissue during in vitro and in vivo processing can depend on the quality of stem cell source. Therefore, the use of different ASC types will be discussed while highlighting the most suitable autologous cells for bio-engineering of skeletal and smooth muscles (Figs. 1 and 2; Tables 1 and 2).

**Table 1:** Stem cell sources for skeletal muscle bioengineering and clinical applications

Stem cell	Origin	Differentiation capabilities	Lineage specific markers	Clinical studies
Satellite cells	Beneath basal lamina of myofibers	Mitotically quiescent stem cells	Pax7 + MyoD-	–
MPCs	Beneath basal lamina of myofibers	Activated upon muscle damage	MyoD + Myf5+	Duchenne muscular dystrophy (DMD) [24], stress urinary incontinence (SUI) [25, 27, 28]
MDSCs	In close proximity to basal lamina of myofibers	Myogenic, chondrogenic and osteogenic	Co-expression of myogenic and endothelial markers	–
MSCs	Bone marrow, fat, skeletal muscle, umbilical cord, blood	Myogenic, chondrogenic, osteogenic and adipogenic	CD105+, CD73+, CD90+, CD45−, CD34−, CD14−, CD3−, HLA-DR	-
ADSCs	Adipose tissue	Myogenic (when cultured in myogenic induction medium containing horse serum)	CD105+, CD73+, CD44+, CD29+, CD90+, CD45−, CD34−	Male urinary incontinence [53, 54], SUI [55]
AFSCs	Amniotic fluid	Myogenic, osteogenic, adipogenic, endothelial, neurogenic and hepatogenic	CD44+, CD73+, CD90+, CD105+, SSEA-4	SUI [58]
HSCs	Bone marrow, skeletal muscle	Myogenic	CD45+/Sca1+	–

**Table 2:** Stem cell sources for smooth muscle bioengineering and clinical applications

Stem cell	Origin	Differentiation capabilities	Lineage specific markers	Clinical applications
ADSCs	Adipose tissue	SMC, urothelium and nerve tissue regeneration	CD105+, CD73+, CD44+, CD29+, CD90+, CD45−, CD34-	–
Endometrial and menstrual blood derived stem cells	Endometrium and menstrual blood	Support urothelial cell growth in vitro	CD90, CD105, CD146 and Oct-4	–
USCs	Urine	SMC differentiation	CD90+, CD105+, CD44+	–

## Skeletal muscle bio-engineering

For cell-based therapy of skeletal muscle damage and diseases, such as surgical and traumatic damage, cancer ablation, congenital defects, degenerative myopathy, volumetric muscle loss, urinary and faecal incontinence, either autologous or allogeneic settings can be used. The autologous therapy is only applicable where the patient has genetically healthy muscle. In the case of genetically impaired muscle cells, such as Duchenne’s muscular dystrophy (DMD), autologous therapy is not an option unless the patient’s cells are genetically modified [8]. An allogeneic stem cell transplant could offer an alternative approach because the stem cell harvest is performed from a donor other than the recipient but immunologically compatible.

### Satellite cells, myoblasts and muscle derived stem cells

Skeletal muscle is one of the adult tissues that still possess the ability to regenerate itself, and this ability resides within a population of cells, defined as satellite cells. These cells have been simultaneously discovered by Mauro and Katz in the early 1960s and have since then been widely accepted as the resident stem cells of skeletal muscles, providing myoblasts for growth, homeostasis and repair [9].

The mitotically quiescent skeletal muscle satellite cells are localized underneath the basal lamina surrounding each myofiber. Disruption of the muscle fiber leads to activation of these cells. Once activated, they divide, on one hand, to maintain a viable satellite-cell pool by self-renewal, and, on the other hand, to produce satellite cell derived myoblasts that further proliferate and finally differentiate before fusing into myotubes [10, 11]. The end-point of differentiation is the formation of mature myofibers [12]. Satellite cells are postnatal cells, committed to the formation of myotubes. They are known to express high levels of the paired box transcription factor 7 (Pax7), which is likely to be involved in supporting satellite cell survival [12, 13]. Furthermore, the process of myogenesis is regulated by a family of muscle-specific transcription factors, expressed in a temporally ordered manner (myogenic differentiation factor 1 (MyoD), myogenic factor 5 (Myf5). MyoD is highly expressed during myoblast proliferation, whereas their differentiation into myocytes is marked by myogenin upregulation. In response to muscle damage, few Pax7 + MyoD- cells return to quiescence whereas the majority Pax7 + MyoD + activated satellite cells commit to differentiation, and fuse with each other to generate new repaired myofiber. Therefore, these activated satellite cells are also referred to as muscle precursor cells (MPC) more recently [14]. Desmin is another protein specific for differentiating myocytes and myotube formation. It is a type III intermediate filament near the Z line of sarcomeres and thus its expression increases towards the terminal differentiation. Typical biomarkers for this end-stage are sarcomeric alpha-actinin and myosin heavy chain 1 and 2 (MyHC). These proteins are the main tools involved in the proper functioning of sarcomeres, leading finally to muscle contraction.

It was demonstrated that human adult skeletal muscle contain, in addition to satellite cells, a population of cells that co-express myogenic and endothelial cell markers. These muscle derived stem cells (MDSCs) showed an ability to regenerate injured skeletal muscle as well as myogenic, chondrogenic, and osteogenic differentiation capacities in vitro [15, 16]. The combination of MDSCs with fibrin-based biomaterials was further assessed by Matthias et al. with regards to volumetric muscle loss injuries and presented promising results [17]. However, it is uncertain whether the MDSCs described by several groups, and isolated by different methods, represent the same stem cell population, or the same population at a different stage of myogenic maturation [18].

The potential use of both the MPCs and MDSCs for therapeutic purposes is recently being highly discussed. The successful implementation of these cells was shown in several pre-clinical studies using pigs [19], dogs [20], mice [21], and human trials [22, 23]. In general, all the clinical studies showed the safety, feasibility and potential efficacy of autologous MPC for the purpose of cell therapy. But due to the use of MPCs and MDCs at different stages the direct comparison of efficacy remains challenging.

One clinical approach uses MPCs for the treatment of DMD by myoblast transfer therapy. Law et al. were able to detect the expression of normal functional dystrophin transcripts within the muscle of DMD patients after transplantation of muscle precursor cells from healthy donors through reverse-transcriptase polymerase chain reaction. They further mention that 81% of the analyzed muscles showed an increase in muscle strength or no continuous muscle loss [24]. However, in multiple other clinical trials, the myoblast transferred did not show any benefit for patients and failed to improve the strength of the patient injected with donor myoblasts [22, 23]. The outcome of various trials hint that several factors come into play which can strongly affect the result of the therapy, such as environmental factors, genetic modifications etc. [8].

Furthermore, the pooled data from 3 phase I/II studies performed through the Cook Myocyte Facility (Pittsburgh, PA, USA) in females with stress urinary incontinence (SUI) showed that the higher the dose of cells injected the better the chance for patients’ responsiveness for sphincter repair [25]. Cook Myocyte assessed safety and efficacy successfully, but also stated that patient population needed to be selected very stringently to allow a reliable comparison between treated and placebo groups [26]. Peters et al. performed treatment on 82 female patients (average age 55 ± 1) with 10, 50, 100 or 200 million cells in 4 ml (applied as 8 × 0.5 ml injections) and followed up 1, 3, 6, 12 months post injection with voiding diaries, incontinence impact questionnaire (IIQ-7) and urogenital distress inventory form (UDI-6). The second dose ranging study by Carr et al. compared low (1, 2, 4, 8 and 16 million cells) vs high (32, 64, 128 million cells) dose groups of in total 38 female patients with an average age of 50 ± 1.6 years. Voiding diaries, pad test and QOF questionnaires (IIQ-7 and UDI-6) were performed over a time of 1, 3, 6, 12 and 18 months. Gerullis et al. reported the largest trial to treat SUI with autologous MPC injections (1.2–19.2 million cells applied in min. 5 injections) in 222 male patients (average age 70) with first clinical improvements showing after 4.7 months [27]. The longest follow up study (purely QOL questionnaire-based) covers 4 years and reports a 75% success rate post cell injection with 0.6–25 million cells (performed circumferentially 9, 12 and 3 o’clock positions), but with a small number of patients [28]. Sharifiaghas et al. report only limited success in treating patients suffering from SUI 24 months after muscle-derived cell injection and mention that multi-centric trials are required to obtain more robust data [29].

**Fig. 3:**
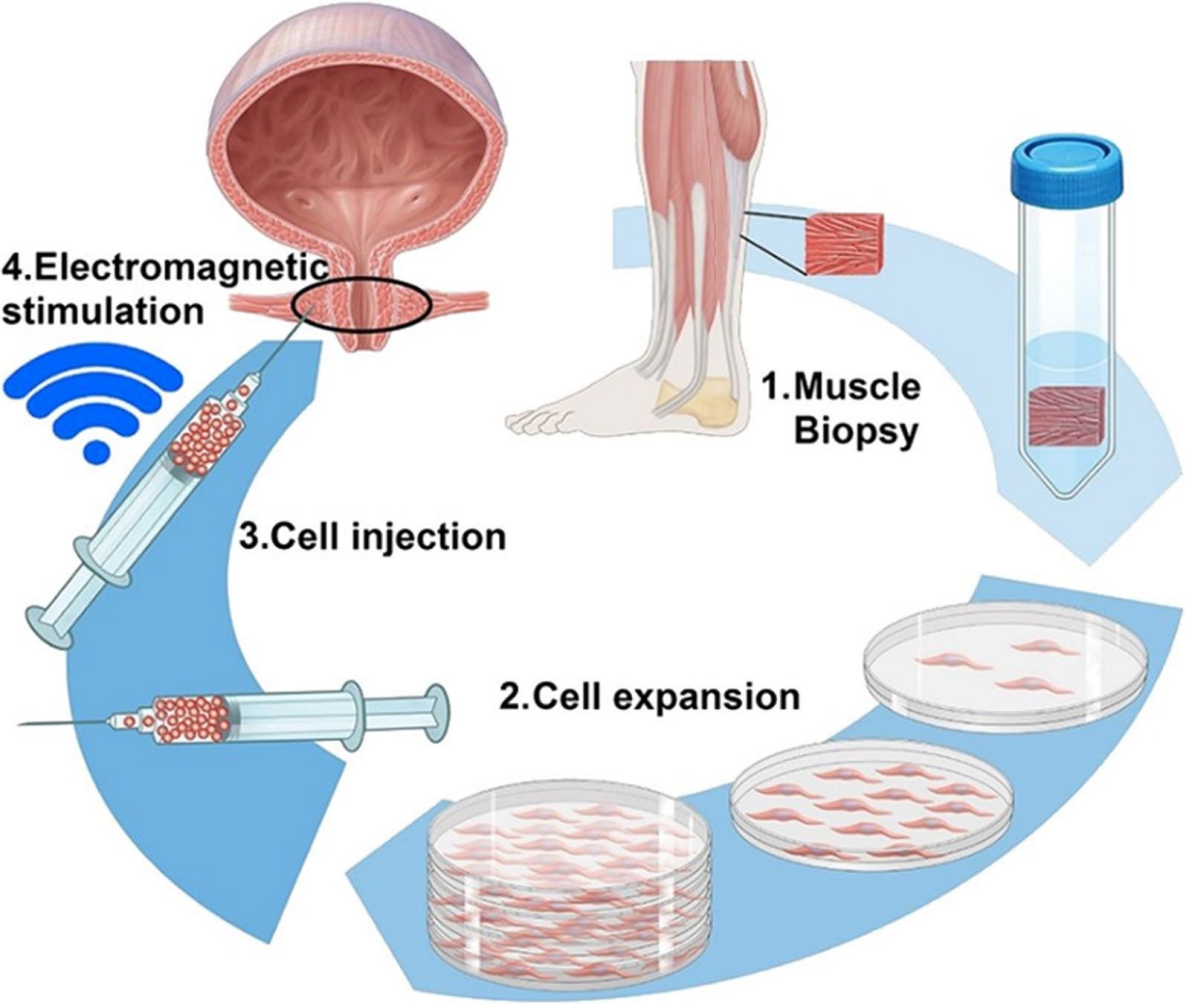
Muscle precursor cells production for clinical trial application of MUSIC project

The safe use of MPCs for the treatment of female patients with incontinence in combination with NMES stimulation has been recently investigated in the Horizon 2020-funded phase I clinical trial MUS.I.C. by the laboratory for urologic oncology and stem cell therapy at the University of Zurich (clinical trial identifier NCT03439527) (Fig. 3). Safety and efficacy of this therapy has to be further assessed in additional clinical trials (e.g. dose-finding). There are few higher phases of ongoing clinical trials, however, the results are not published yet. In all these studies the improvements were based on quality of life assessments or in the case of incontinence on urodynamic tests after cell therapy [28].

If successful, the same strategy can directly be translated to a variety of muscle diseases, e.g. insufficiency of other sphincter muscles, vocal cord dysfunction or regeneration of the smaller eye muscles. This application field for MPCs was verified in a phase I/IIa clinical study supporting the hypothesis of the safety and efficiency of local injection of autologous myoblasts in the pharyngeal muscles in patients with oculopharyngeal muscular dystrophy [30]. One of the main challenges of the cell therapy is precise and minimally invasive delivery of cells. There are several ways to inject the cells to the right location. Transurethral ultrasound-guided injections of autologous cells isolated from limb skeletal muscle biopsies were so far the method of choice by several groups [31, 32]. This method is also standard for the injection of bulking agents like collagen in the clinical practice [33]. Ultrasound guidance was also used to monitor percutaneous trans-coronary-venous transplantation of autologous myoblasts in infarcted myocardium [34]. Recently magnetic resonance imaging (MRI) raises attention as a useful tool for guidance during injection of drugs and potentially of cells [35] Pulsed focused ultrasound is a new ultrasound technique that associated with magnetic resonance guidance was recently suggested as a new imaging modality that may be utilized to target cellular therapies by increasing homing to areas of pathology [36]. Overall, the most successful deliveries of myogenic cells have been done either operatively in 3D scaffolds or in collagen carrier that facilitates cell settling into the new cell niche.

However, the next challenge in this regeneration chapter will be the treatment of larger muscles.

### Mesenchymal stem cells

Human adult mesenchymal stem cells (MSCs) are multipotent and their ease of isolation and potential for differentiation make them great candidates for the use in TE. The different in vivo distribution of MSCs includes bone marrow (BM), adipose, synovial membrane, skeletal muscle, periosteum, dermis, pericytes, blood, human umbilical cord, lung and dental pulp [37]. Currently, independent of their origin these cells are called MSCs. They are spindle shaped plastic- adherent cells with the surface marker phenotype of cluster of differentiation (CD): CD105+, CD73+, CD90+, CD45−, CD34−, CD14−, CD3− and HLA-DR. MSCs can differentiate into several cell types, including chondrogenic [38], osteogenic and adipogenic lineages in vitro. MSC are known to be immunosuppressive and immune privileged cells. Myogenic differentiation of bone marrow derived human and mouse MSC was shown in several studies [39, 40]. Ferrari et al. demonstrated that BM-derived MSC from transgenic mice could be recruited to injured muscle and help in the regeneration of damaged fibers [40]. Dezawa et al. confirmed that these cells can differentiate to skeletal myogenic lineage, when treated with basic fibroblast growth factor (bFGF), platelet derived growth factor-AA, and neuregulin [41]. The ability of BM-MSCs to contribute to skeletal muscle regeneration has been presented in an injured model of tibialis anterior muscle and revealed that BM-MSCs support the repair and regeneration 4 months after transplantation [42]. However, the myo-regenerative capacity of BM-MSCs seemed to be lower in comparison to synovial membrane-MSCs and adipose tissue- derived MSCs [42]. It was demonstrated that multipotent synovial membrane-derived adult human MSCs can be isolated and induced to develop to chondrogenic, osteogenic, myogenic, or adipogenic lineages in vitro regardless of donor age and cell passage number [43].

In a pig model for severe radiation burn, muscle regeneration was assessed 1 year after skeletal muscle surgery with and without bone-marrow MSC treatment [44]. The bone-marrow MSC treatment improved regeneration substantially compared to the purely surgical intervention. A combinational approach was tested in a rat model by encapsulating bone-marrow MSCs in microbeads to treat muscle loss injury and lead to a reduced regeneration time compared to the sham treated group, which was only capable of incomplete repair [45]. Although several studies showed successful preclinical use of MSCs in animal models of various diseases, substantial challenges still need to be overcome before MSC therapy can be used in clinical practice. Since MSCs have been reported to promote tumor growth and metastases [46] particular attention should be paid to the biosafety of using MSC for clinical applications [47]. There are no reports yet on the clinical application of MSCs for skeletal muscle engineering.

### Adipose derived stem cells

Adipose tissue is an abundant and accessible source of stem cells with multipotent characteristic. Hence, adipose derived stem cells (ADSCs) bear the potential for use in TE applications because they are easily accessible, abundantly available and they can be isolated from adipose tissue and adipose aspirates [48]. There are several reports displaying that ADSCs can differentiate to the myocyte lineage when cultured in myogenic induction medium containing horse serum [49]. Induced ADSCs express MyoD and myogenin, the main transcription factors regulating skeletal muscle differentiation [50]. According to the histological results, ADSCs can fuse and form multinucleated myotubes in vitro and can be enhanced under the influence of biophysical stimulation [51]. However, up to now, no animal and clinical studies using ADSC for skeletal muscle myogenesis have been reported showing substantial participation of these cells in skeletal muscle repair. A pure paracrine effect of ADSCs was postulated in a study where these cells were injected into the soleus muscles of female rats. Increased muscle repair and force was observed after 2 weeks upon injection. However, no significant differences were observed after 4 weeks compared to control group suggesting a paracrine mechanism of action [52]. The injection therapy with ADSCs was described as a potentially safe method to treat male urinary incontinence [53, 54]. In addition, a similar clinical study was performed to find out whether transurethral injections of autologous ADSCs with collagen could be safe and effective for SUI treatment in 5 females. In this study, the injection of 2.4-4 ml containing 2.5–8.9 million autologous ADSCs (passage 3–4) in collagen gel and saline was confirmed to be safe and well tolerated, but feasibility and efficacy was suboptimal [55].

### Amniotic fluid derived and umbilical cord blood stem cells

In 2003, Prusa et al. demonstrated that the amniotic fluid could contain pluripotent stem cells, which were positive for the nuclear transcription factor octamer transcription factor 4 (Oct4). Oct-4 is a marker for pluripotent human stem cells and known to be expressed in embryonic stem cells and embryonic germ cells [56]. The amniotic fluid stem cells (AFSC) are multipotent and have been displayed to differentiate into adipogenic, myogenic, endothelial, osteogenic, neurogenic and hepatogenic lineages. The AFSCs showed expression of the myogenic lineage-specific markers, MyoD and desmin, when cultured in myogenic-specific induction media [57]. However, to date, only limited clinical studies were reported showing the use of AFSCs for muscle regeneration/repair. Chun et al. reported that human AFSCs present an accessible source for muscle regeneration and injected cells did not induce teratoma formation and immunogenicity [58].

Lee et al. reported the use of human cord blood stem cells injection for the treatment of stress urinary incontinence. The postoperative urodynamic study showed improvements already after 3 months of stem cell injection [59]. In this group, 39 female patients with an average age 51 ± 8 years received 2 injections of 430 ± 190 million cells/2 ml at a 4 and 8 o’clock position. Follow up and evaluation was performed after 1, 3 and 12 months using voiding diaries, quality of life (QOF) and other questionnaires and showed that umbilical cord blood derived stem cells can be effectively used for all types of incontinence.

### Bone marrow derived hematopoietic stem cells

The differentiation potential of hematopoietic stem cells (HSCs) to skeletal muscle has been previously reported [60], but the related molecular mechanism remains elusive. Numerous studies described that whole bone marrow or population of HSCs (CD45+) and mononuclear myoblasts are able to give rise to muscle fibers [61]. From muscle isolated CD45+/Sca1+ (stem cells antigen 1) cells can form myogenic clones when co-cultured with skeletal myoblast cells in vitro [62]. In a similar approach, it was shown that CD45+/Sca1− cells derived from muscle, showed in vivo myofiber-forming ability but were not able to differentiate into myocytes neither alone, nor in co-culture with skeletal myoblasts in vitro. Furthermore, it was demonstrated that CD45+/Sca1+ cells derived from bone marrow and muscle did not express main myogenic markers like Pax7 and MyoD, although they underwent myogenic reprogramming and participated in myofiber fusion. These results suggest that CD45+/Sca1+ cells isolated from muscle form a population that contributes to muscle tissue regeneration but is not to be mistaken with the original muscle satellite cells population [63]. Many factors, such as IL-4 and IL-6, are influencing the cell fusion between bone marrow derived stem cells and myoblasts [64]. Several efforts were made to characterize mechanisms of in vivo influence of bone marrow-derived cells to myofibers and it appeared that HSC participated in muscle regeneration by direct fusion with myogenic cells [65]. As it was described in a single-cell transplantation study, HSCs derivatives that integrate into regenerating muscle fibers exist in the pool of hematopoietic cells known as myelomonocytic progenitors [66]. Mouse studies showed the participation of bone-marrow derived cells in the composition of an intact satellite cell niche as well as during regeneration [67]. Transplantation of HSCs in dystrophic dogs, however, did not restore dystrophin expression [68]. To date, the use of bone-marrow derived stem cells in various muscle disease therapy models is rather limited.

## Smooth muscle bio-engineering

Smooth muscle tissue is an involuntary, non-striated muscle with neural innervation from the autonomic nervous system. Smooth muscle (SM) is a functionally critical component of a variety of tissues and any attempt to engineer these tissues must include the development of functional smooth muscle cells (SMCs) with a contractile phenotype.

### Smooth muscle cells

SMCs are an essential cell type found in several organs, including the respiratory tract, gastrointestinal tract, urinary bladder, uterus, male and female reproductive tracts, and the vascular system. One of the main characters of SMCs is their contractility, which plays an important role in angiogenesis, blood pressure maintenance, and mechanical regulation of hollow organs. SMC contraction is regulated by the activation of myosin and actin, and the calcium ions (Ca^2+^) which serve as the initiator of the contraction [69]. SMCs contractile phenotype is characterized by high expression of specific contractile proteins including smooth muscle actin (SMA), calponin, *h*-caldesmon, SM22, smoothelin, and smooth muscle myosin heavy chain [70]. One of the major problems in engineering SMCs is finding a reliable source of healthy SMCs that can be easily and safely harvested with minimal harvest site morbidity. Several groups have reported the use of SMCs isolated directly from biopsies of the diseased organs, such as bladders or vessels [71]. Using the biopsies from a target organ may have several problems including morbidity caused by complex surgery and limited sample size. In addition, the SMCs derived from diseased organs possess and sustain pathological characteristics in vitro [72]. Prominently, the mature SMCs isolated from healthy sources show limited proliferation capacity and usually lose their contractile phenotype during their in vitro proliferation and expansion [73]. SMCs were isolated by explant and enzymatic digestion techniques from human and rodents’ bladder tissue [74]. However, in contrast to mentioned reports, in a pre-clinical study, it was shown that tissue engineered muscle from normal and diseased human bladders keep their phenotype in vitro and after implantation in vivo in athymic mice [75]. This study suggested that there were no phenotypic or functional differences between muscle cells obtained from urodynamically normal or neurogenic bladders. In a clinical study of the same group, patient`s own SMCs along with urothelial cells were used to generate bladders that were implanted in patients requiring cystoplasty. The implanted engineered bladders showed improved functional parameters over five years [76]. In spite of these few reports, it has been shown that SMCs derived from diseased organs, retain and sustain their pathologic characteristics in vitro [72]. This may affect the regenerative ability of the newly engineered tissue. To overcome this problem other cell sources or, if possible, genetic corrections are necessary for the engineering of SMCs. The most promising candidate of adult stem cell sources for SMC bioengineering are, ADSCs, bone marrow derived MSCs, urine derived stem cells, endometrium cells and menstrual blood cells.

### Adipose derived stem cells

ADSCs are one of the most promising ASCs, which can be easily and efficiently differentiated to SMCs for engineering hollow organs and vessels. Efficient myogenic differentiation of ADSCs was reported by several groups using smooth muscle inductive medium containing MCDB131, supplemented with fetal bovine serum and heparin [77]. It was shown that under the effect of transforming growth factor-B1 and bone morphogenetic protein 4, both early- and mid- differentiation markers (α-SMA, SM22a, calponin), as well as a late marker (SM myosin heavy chain) of SMC differentiation were identified [78]. The ADSCs derived from different sites show different myogenic differentiation abilities in vitro*.* In a comparative study of ADSCs derived from different sites in rabbit, the adipose tissues of the nape of the neck of rabbit was found to be the most suitable source for engineering the lower urinary tract [79]. Similarly, it was demonstrated that subcutaneous adipose tissue has higher differentiation capability than omental adipose tissue [80]. In an animal study, bladder acellular matrix grafts seeded with ADSCs showed bladder SMCs, urothelium and nerve tissue regeneration [81]. Another study demonstrated that polylactide acid scaffold seeded with ADSC showed a normal urethral architecture with a thickened muscle layer compared to unseeded scaffold control group [82]. In a more recent study, Tremp et al., established a reliable small animal model for hypocontractile bladder and demonstrated that ADSCs support the early restoration of bladder voiding with improved voiding pressures and molecular expression of SMC contractile proteins after cell therapy [83].

Although partial or complete regeneration of SMCs in cell seeded grafts are described, improved functionality is still not shown in any animal study and requires further investigation.

### Endometrium and Menstural blood derived stem cells

The endometrium is known to be a highly regenerative tissue and was reported to be a source for mesenchymal stem cells [84]. MSCs can be harvested from the endometrium by two methods: an endometrial biopsy from the uterine or collection of menstrual blood [85]. In contrast to bone marrow and adipose tissue, which require at least local anesthesia, the obtainment of these cells does not require an anesthetic procedure [86]. Both endometrium and menstrual blood stem cells are similar to bone marrow and adipose derived MSC and share similar lineage specific markers CD90, CD105 and CD146 but are exceptional in the expression of octamer-binding transcription factor 4 (Oct-4) [87]. Endometrial stem cells could differentiate into SMCs, thus making them an attractive cell source for building organs such as urinary bladder wall and for repairing the pelvic floor in females [88]. However, the SMCs differentiation potential of human endometrial-derived stem cells has not been demonstrated in any in vivo pre-clinical or clinical study.

### Urine derived stem cells

Currently, urine derived stem cells (USCs) were isolated from voiding urine and are suggested as a good non-invasive cell source for urological tissue reconstruction when the cells are isolated within 24 h after urine collection [89, 90]. The USCs possess biological characteristics of MSCs and show similar cell surface marker expression profiles [91]. It was demonstrated that the voided USCs originate from the kidney, because cells obtained from women who had received transplanted kidneys from male donors contained the Y chromosome and expressed normal renal cell markers (PAX2 and PAX8) [92].

When USCs were induced in myogenic medium, they expressed all SMC lineage specific markers calponin, smoothelin and SMA [89]. In addition, myogenic differentiated USCs showed contractile function that is comparable to SMCs [90]. Furthermore, myogenic differentiated USC could form multiple layers of SMCs when implanted subcutaneously in a nude mice model [93]. Because they originate from the urinary tract system, USCs are suggested as a good stem cell source for bladder tissue engineering. Additional benefits are that they can be collected using a simple, safe, low-cost and non-invasive technique and can be differentiated efficiently to bladder SMCs [92, 93]. USCs are highly expandable and do not induce teratomas or tumors in vivo. Furthermore, preclinical studies of cell therapy with USCs showed positive outcome in models of stress urinary incontinence [94], urethra and bladder reconstruction [93, 95]. Moreover, human USCs seeded scaffold-heparin-bFGF grafts exhibited enhanced biocompatibility, increased bladder capacity and compliance, signified by smooth muscle and urothelium layers in a partial cystectomy rat model [96]. Until now, no clinical studies were reported using USCs for tissue regeneration or repair.

### Outlook: future towards better muscle regeneration

Substantial progress has been made in the field of cell therapy for muscular disorders. Multiple cell types were being discovered for cell therapy, each presenting a great therapeutic potential. As tumorigenicity and ethical concerns seem to hinder the use of embryonic stem cells, ASCs possibly offer a feasible alternative and pave the way towards clinical translation of the proposed therapies. The source of the stem cells can affect the procedure for ultimate clinical application of the wanted tissue. The engineered muscle tissue must be customized to the needs of the individual tissue, aiming for the improvement of the contractility and assurance of the physical function. Phenotypically similar stem cells may behave differently, and phenotypically different cell types may differentiate towards the same tissue type, depending on the microenvironment in vivo. Additionally, genetic corrections of isolated cells may be needed in the future as a combinational approach of cell and gene therapy for the continuous development towards personalized medicine. The ability of ASCs to form muscle tissue decreases with age and disease. Aging affects the regenerative ability of muscle by reducing both stem cells pool and functionality [97]. The changes which occur in the microenvironment of the muscle niche during aging could be a main contributor to the functional decline in muscle stem cells [98]. Therefore, improvement of microenvironment or bioengineering better stem cell quality may turn back the clock on aging muscles.

## Conclusions

For skeletal muscle tissue engineering, satellite cells were initially considered to be the best candidate for cell therapy. However, they are challenging to expand in vitro, generating an insufficient number of cells for tissue-engineering purposes. Therefore, MPCs which are formed after activation of satellite cells, are now suggested to be the most suitable cell-source for skeletal muscle engineering. They can be easily isolated and efficiently expanded in vitro*.* Therefore, MPCs, are extensively used in clinical trials [27, 30]. Nevertheless, previous clinical trials with MPCs showed variable cell numbers, different injection modes and the use of different substances, and possibly therefore led to variable outcomes. The next important point is the microenvironment for the non-muscle derived stem cells [1].

For smooth muscle bioengineering, the multi-lineage capacity and availability of ASCs make them the best candidates for reconstruction of human smooth muscle containing tissues and organs. While great advancements have been made in adult stem cell-induced tissue engineering and their myogenic differentiation, future studies are needed to highlight effective seeding techniques and methods to generate the ideal contractile and dynamic muscle.

In addition, safety remains one of the main concerns in cell therapy and regenerative medicine. The production of safe cell products requires tightly regulated process to ensure the cells maintain their phenotype, functional potential, and remain unchanged as well as clear of any microbiological contaminations. Therefore, a strict quality control system for cell production must be applied to assure the safety and efficiency of the final products of cell therapies.

## Data Availability

Not applicable.

## Abbreviations

ASCs: Adult stem cells
TE: Tissue engineering
MPCs: Muscle precursor cells
HSC: Hematopoietic stem cells
ADSC: Adipose derived stem cells
MSCs: Mesenchymal stem cells
SMCs: Smooth muscle cells
USCs: Urine derived stem cells
SMA: Smooth muscle actin
